# The Role of the Intestinal Context in the Generation of Tolerance and Inflammation

**DOI:** 10.1155/2012/157948

**Published:** 2011-09-22

**Authors:** Bernardo Sgarbi Reis, Daniel Mucida

**Affiliations:** Laboratory of Mucosal Immunology, The Rockefeller University, 1230 York Avenue, New York, NY 10065, USA

## Abstract

The mucosal surface of the intestine alone forms the largest area exposed to exogenous antigens as well as the largest collection of lymphoid tissue in the body. The enormous amount of nonpathogenic and pathogenic bacteria and food-derived antigens that we are daily exposed sets an interesting challenge to the immune system: a protective immune activity must coexist with efficient regulatory mechanisms in order to maintain a health status of these organisms. This paper discusses how the immune system assimilates the perturbations from the environment without generating tissue damage.

## 1. Interface between the Outside and Inside Environments

The intestinal mucosa forms the largest area of the body in direct contact with the exterior environment. If expanded, the surface of the small intestine alone can reach roughly the size of a tennis court, or 100 times the area of the skin [1]. In the skin, several layers of cells, including stratified epidermis, and dermis, generate a physical barrier that separates the internal components of the body from the outside. On the other hand, in the intestine, a single layer of absorptive epithelial cells forms an interface between the lumen (outside environment) and the lamina propria (inside environment). If one sees our body as a target for attack from infectious and pathogenic organisms, the structure of intestinal epithelia is counterintuitive, since the intestine is exposed to constant colonization by bacteria and is a host to an enormous quantity and diversity of microbes, including commensals and potential pathogens. More than 100 trillion microbial cells colonize the human gut, which amounts to ten bacteria for every one human cell. The vast majority of these bacteria are not pathogenic, but rather perform a variety of beneficial functions to the host [2]. A recent study, using extensive Illumina sequencing of fecal DNA samples, estimated that the human microbiome contains more than 1000 bacterial species, with more than 160 different species generally present in each person [3]. These results highlight a high degree of person-to-person variation, possibly influenced by a distinct host genetic landscape and environmental conditions. 

Other mucosal surfaces also harbor a diverse microbiota. For instance, over 200 genera of bacteria were identified in a human skin microbiome study [4]. However, the intestinal mucosa is peculiar since it has to deal with intense bacterial colonization and at the same time absorption and digestion of nutrients. In that regard, it should be noted that the large intestine contains most of the microbiota while the small intestine is the main place for absorption and digestion of nutrients. 

In addition to the exposure to innocuous antigens, the intestine is also a place where many different types of infections can occur, including infection by viruses, bacteria, parasites, and fungi. Commensal bacteria, generally involved in symbiotic interactions with the host, have also been correlated with the development of inflammatory bowel diseases such as ulcerative colitis and Crohn's disease. Similarly, dietary proteins can trigger food allergies and celiac disease. Therefore, it is reasonable to argue that the great majority of processes in the gut are not generated towards “defense” against invading organisms, but are rather a consequence of chronic exposure to large amounts of harmless and often beneficial antigens. This scenario poses an interesting challenge to the immune system, since most of the “nonself” interactions should probably be tolerized as “self”. How does the immune system associated with the intestine influence and assimilate the perturbations from the environment without generating pathology? 

## 2. The Immune System at the Intestinal Interface

As expected, the intestinal mucosa is filled with a diverse and large number of immune cells. The gut-associated-lymphoid-tissue (GALT) includes the Peyer's patches (PP) and isolated lymphoid follicles (ILFs). However, most of the immune cells in the intestine are associated with the intestinal villi, either in the intraepithelial or lamina propria compartments, which are the focus of this paper. Estimations based on histological sections indicate that there are more T cells in the intraepithelial compartment alone than in the spleen [5]. Moreover, the cells in the intestinal mucosa consist of mainly activated or antigen experienced T cells (CD45RB^lo^, CD44^hi^, CD69^hi^, CD62L^lo^) that are capable of producing several proinflammatory cytokines such as IL-4, IFN-*γ*, IL-17A/F, IL-22, and TNF-*α* [6–15]. 

The intraepithelial compartment of the intestine is unique in regards to its lymphocyte populations. Most of intraepithelial lymphocytes (IELs) are CD8*αα*-expressing TCR*αβ* and TCR*γδ*, with CD8*αα*-CD4 and -CD8*αβ* TCR*αβ* cells and double-negative T cells contributing in lower numbers. While CD8*αβ* and CD4 IELs are rare early in life, these populations steadily increase with age likely as a consequence of exposure to exogenous antigens [16–19]. IELs also express natural killer (NK) cell receptors, both activating and inhibitory, which allow these cells to change their resting state to a cytotoxic and potentially inflammatory state [7, 9, 20–23]. The development and function of IELs have been recently reviewed elsewhere [24] and will not be the focus of this paper.

Contrary to the IELs, lamina propria lymphocytes (LPLs) contain T and B cell populations with similar frequency to peripheral lymphoid tissues. Additionally, lamina propria B cells produce large amounts of immunoglobulin (Ig) molecules, mostly belonging to the IgA isotype, which is the most abundant antibody isotype in the body. Furthermore, LPL cells reside among several types of antigen presenting cells (APCs) and other types of innate immune cells, so-called innate lymphoid cells, that can function to either promote or suppress inflammation [25–28]. IELs, LPL, dendritic cells (DCs), and intestinal epithelial cells are in constant interaction and their cross-talk is reinforced by cell surface receptor-ligand interactions, including *α*4*β*7/MadCAM, *α*_E_ *β*7(CD103)/E-cadherin and CD8*αα*/TL contact [29–31]. Through the expression of tight junctions, epithelial-associated CX_3_CR_1_^+^ APCs are able to establish contacts with the neighboring epithelial cells, while extending their dendrites to sample luminal antigens, including whole bacteria [32]. Both APCs and epithelial cells express toll-like receptors (TLRs) that induce cellular activation and lead to the migration of DCs to regional lymph nodes, where they can present processed antigens to the naïve T cells. The diversity of functions exerted by innate immune cells in the intestinal lamina propria is also achieved through the production of several different cytokines and other soluble factors such as IL-22, IL-23, and retinoic acid [25–28]. Consistently, high frequency of proinflammatory Th17 cells and regulatory T cells can be found in the lamina propria of the small and large intestine, respectively [33–35]. 

Recent advances in mucosal immunology research contributed to our understanding of how the intestinal context [36] plays that critical role in the balance between protective immune responses and tolerance to harmless antigens. 

## 3. The Intestinal Context: Microbiota

Commensal microorganisms actively interact with the absorptive intestinal mucosa and influence the basal activity of the immune system as well as the amplitude of the immune response. The importance of the microbiota to the development of the host immune system is evident in germ-free animals (born and raised in completely aseptic conditions). The development of the local or systemic immune system is defective in germ-free mice. For example, germ-free mice show reduced germinal-centers in the spleen and reduced systemic IgG and IgA antibodies [2, 37, 38]. These mice have fewer and smaller Peyer's patches, reduced mesenteric lymph nodes, decreased cell numbers, and virtually no IgA production in the lamina propria relative to conventional animals [37, 39]. IELs are also compromised in germ-free animals, particularly TCR*αβ* IELs, with a drastic decrease in cell number and cytotoxic capacity throughout the intestine [36, 40]. 

Similarly, the microbiota is able to modulate the activity of innate immune cells, including APCs and innate lymphoid cells, in the lamina propria [28, 41]. Commensal bacteria-derived ATP has been shown to directly activate lamina propria CD11c^low^CD70^high^ cells to produce IL-6, IL-23, and TGF-*β* and induce local differentiation of Th17 cells [42]. The reduced amount of ATP in germ-free animals was proposed [42] as an explanation to the depletion of Th17 cells in the lamina propria of these mice [33, 34, 42]. Conversely, recent studies found that the commensal *segmented filamentous bacteria* (SFB) is present in mouse colonies with a high frequency of IL-17-producing cells in the intestine [33, 34]. While germ-free mice lack Th17 cells in the lamina propria of the small intestine, the mono-colonization of germ-free mice with SFB restores the number of Th17 cells to conventional levels. In addition, no change in Th1 cells was observed indicating that SFB induces differentiation of CD4 T-cells into Th17 cells. However, no association between SFB colonization and ATP levels was reported [33, 34]. The IL-23/IL-17 axis of the CD4 LPLs exert a protective function against extracellular pathogens while being detrimental in different models of inflammatory bowel diseases [43]. Interestingly, the SFB-induced Th17 responses also enhance susceptibility to systemic autoimmune disorders such as arthritis and experimental autoimmune encephalomyelitis (EAE) [44, 45]. 

A contrasting example of a bacterial metabolite that contributes to the mucosal immunity is the short-chain fatty acids (SCFAs), which is produced by fermentation of dietary fiber by *Bifidobacterium* [46]. SCFAs bind to G-protein-coupled receptor 43 (GPR43, also known as FFAR2) and inhibit inflammatory responses during DSS-induced colitis by suppressing the differentiation of IL-17 producing cells in the lamina propria of conventional mice, suggesting that germ-free mice are more susceptible to this model of colitis due to reduced SCFA in the intestinal environment [46].

The commensal bacteria *Bacteroides fragilis* is also associated with suppression of Th17 and other inflammatory responses in the intestine by expression of polysaccharide A (PSA) via IL-10 production [47, 48]. A recent study showed that PSA is recognized by TLR2-expresing T cells and promotes their production of IL-10 [49]. Furthermore, PSA-treated Tregs are more efficient at suppressing activated T-cells *in vitro*, and *Bacteroides fragilis* mono-colonized recipient-mice induce higher numbers of Treg cells and show reduced Th17 responses after naïve CD4 adoptive transfer [49]. In addition to *Bacteroides fragilis*, a recent report by Atarashi and coworkers identified *Clostridium* spp., a genus of gram-positive bacteria, belonging to the Firmicutes phylum, as a major inducer of Tregs in the colon of conventional mice [35]. The authors showed a specific depletion of the induced-Treg (iTreg) population in the colon of germ-free animals, and mono-association with 46 different species of *Clostridium*, but no other classes of bacteria, completely restored this population [35]. These results show that microbial-derived mechanisms can affect both innate and adaptive immunities and promote immune-regulation in the intestinal surface.

In the same vein, several studies have documented alterations of gut microbiota (dysbiosis) in patients with IBD [50–54]. Frank et al. described a case control study of the intestinal microbial ecology in IBD and non-IBD controls where they found a marked decrease in the representation of two prominent constituents of the gut microflora, Bacteroides and Lachnospiraceae, in the IBD-specific group compared to controls [55]. However, there remains to be established a cause-effect relationship between dysbiosis and IBD. Furthermore, it is not clear how immune activity affects the composition of the commensal bacteria in health and disease. These results highlight the crucial role that the microbiota play in the homeostasis of intestinal immunity.

## 4. The Intestinal Context: Diet

Although much of the focus in mucosal immunology in recent years was given to the microbiota, most of the immune system antigen interaction in the gut is associated with the small intestine, where the nutrients are absorbed. The majority of these antigens readily get access to the immune system through the intestinal epithelia, M cells, or direct sampling by CX_3_CR_1_-expressing lamina propria macrophages and probably additional APCs. 

Beside the microbiota, the exposure to food proteins has also been shown to play a crucial role in the development and maintenance of the intestinal immune system [56] as well as in susceptibility to systemic infection [57]. The importance of food proteins to systemic immunity can also be appreciated by the fact that we ingest around 100 grams of protein daily, and up to 0.5% (500 mg/day) of ingested proteins can be found intact in blood circulation a few hours after ingestion [58]. 

The relevance of dietary proteins in the maturation of the immune system was demonstrated by elegant studies in “antigen-free” mice (germ-free mice fed an elementary diet). The studies reported a marked reduction in the lymphocyte populations in the gut in such an antigen-deprived environment, with further reduction in systemic immunoglobulins (IgG and IgA) when compared to germ-free animals [59–61]. However, both the repertoire and total production of IgM is maintained in antigen-free animals, suggesting that the immune system has a basal or natural level of activity independent of exogenous antigens [62]. 

Similarly to the microbiota, food proteins are potentially immunogenic and help to maintain the “immunological tonus”. Nevertheless, in general, the exposure to dietary antigens does not generate pathological responses. Indeed, mucosal exposure to antigens efficiently inhibits the development of immune responses to subsequent challenges with the same antigen, a phenomenon described as oral tolerance [63, 64]. It was demonstrated that peripheral generation of Foxp3-expressing Treg cells by TGF-*β*  [65] is a crucial event in oral tolerance induction in mice harboring monoclonal repertoire by both B and T cells (TBmc). Moreover, using the same experimental model, Curotto de Lafaille et al. showed that lack of functional Foxp3 results in abrogation of oral tolerance induction [66].

Recent studies have further elucidated induction and effector phases of oral tolerance. It is thought that antigen-sampling by lamina propria APCs (including CX_3_CR_1_ resident macrophages) followed by antigen-transporting and presentation by migratory CD103^+^CCR7^+^ in the MLN is crucial for generation Foxp3-expressing Treg cells oral tolerance induction [27, 65–69]. Hadis and coworkers have also shown that after Treg cell induction in the MLN, their migration to the lamina propria and expansion mediated by CX_3_CR_1_ resident macrophages is essential for the effector phase of oral tolerance [68].

In addition to their regulatory role, it was also demonstrated that mucosal DCs from mesenteric lymph nodes (MLNs) and Peyer's Patches (PPs) are unique in their capacity of degrading vitamin A to generate retinoic acid (RA) [70]. RA, in a TGF-*β*-dependent process, was proposed to play a crucial role in iTreg induction [25, 27, 69], demonstrating that diet-derived factors are also part of immune regulatory mechanisms involved in the prevention of aberrant immune responses towards the diet itself and other environmental antigens. 

When oral tolerance is abolished, inflammatory processes generally arise resulting in the development of food allergies and other diseases. An example of food-related gut disorder is celiac (or coeliac) disease (CD), a condition that damages the lining of the small intestine and prevents it from absorbing nutrients. The damage is due to a lack of tolerance to gluten, a group of proteins found in wheat, barley, rye, and possibly oats. The break in tolerance leads to an exacerbated (mostly) Th1 immune response to specific gluten antigens (gliadin) in the small intestine after ingestion of gluten (reviewed by [71]). The pathogenic immune response in celiac disease is dependent on antigen (gluten peptides) presentation via major histocompatibility complex (MHC) class II molecules to CD4 positive T-cells. Celiac disease is strongly linked to genetic predisposition found in individuals expressing the MHC- or human leukocyte antigen- (HLA-) DQ2, DQ8, or DQ2/8 and the absence of these human HLA alleles rules out the diagnosis of celiac disease in suspected patients. These MHC class II molecules can be induced in intestinal epithelium under inflammatory conditions and are able to efficiently bind and present gluten peptides, activating proinflammatory CD4^+^ effector T cells [71]. The genetic background together with the intestinal context, in which the gluten protein is presented to T-cells, are the main factors in the balance between tolerance and inflammation in CD development. IFN-*γ* and IL-21 double-producing CD4^+^ T cells [72] and also Th17 cells [73] have been implicated in celiac disease as well. The differentiation of pathogenic, rather than regulatory, CD4^+^ T cells is thought to be induced by proinflammatory cytokines, including IL-15 and IFN-*α*, that are present in the intestinal mucosa from celiac disease patients [71]. One of the mechanisms proposed for IL-15 function in CD is the disruption of TGF-*β*-mediated signaling through SMAD3 [74]. Additionally, an elegant study by DePaolo and coworkers reported that IL-15 may also synergize with retinoic acid to enhance inflammatory responses and CD-like inflammation [75]. It should be noted that this is in sharp contrast to the anti-inflammatory effects of retinoic acid in conjunction with TGF-*β* reported above, which include induction of Treg cells and suppression of inflammatory Th17 differentiation [25]. These results demonstrate how one metabolite may have strikingly different effects depending on microenvironment milieu and cell target of this metabolite. They also point out how deleterious are the consequences when the robust mechanisms of tolerance induction in the mucosal surfaces are broken.

## 5. Concluding Remarks

The dilemma faced by the mucosal immune system to induce tolerance to antigens (rule) or to engage an inflammatory immune response (exception) is daily dealt with through multidirectional interactions between the immune cells and environmental factors that permeate the mucosal surfaces. The identification of cellular and molecular mechanisms involved in this process will likely contribute to new approaches for prevention and treatment of inflammatory bowel diseases (IBD) and also other systemic inflammatory diseases.
